# The effects of insulin on the inflammatory activity of BV2 microglia

**DOI:** 10.1371/journal.pone.0201878

**Published:** 2018-08-27

**Authors:** Fiona Brabazon, Sara Bermudez, Michael Shaughness, Guzal Khayrullina, Kimberly R. Byrnes

**Affiliations:** 1 Neuroscience Program, Uniformed Services University of the Health Sciences, Bethesda, MD, United States of America; 2 Department of Anatomy, Physiology and Genetics, Uniformed Services University of the Health Sciences, Bethesda, MD, United States of America; 3 Center for Neuroscience and Regenerative Medicine, Uniformed Services University of the Health Sciences, Bethesda, MD, United States of America; National Institutes of Health, UNITED STATES

## Abstract

Microglia are the macrophages of the central nervous system (CNS), which function to monitor and maintain homeostasis. Microglial activation occurs after CNS injury, infection or disease. Prolonged microglial activation is detrimental to the CNS as they produce nitric oxide (NO), reactive oxygen species (ROS) and pro-inflammatory cytokines, resulting in neuronal cell dysfunction and death. Microglial activation is implicated in the neurological deficits following traumatic brain injury (TBI) and Alzheimer’s disease. Intranasal insulin administration is a promising treatment of Alzheimer’s disease and TBI. However, the exact effect of insulin on microglia is currently unclear. The goal of this study was therefore to examine the effect of insulin administration on activated microglia. The microglial cell line BV2 were exposed to a pro-inflammatory stimulus, lipopolysaccharide (LPS), followed by insulin administration. Outcome measures were conducted at 24 hours after treatment. *In vitro* assays quantified NO and ROS production. Western blot, immunocytochemistry and phagocytosis assay further examined the effect of insulin on microglial activity. Insulin treatment significantly reduced NO, ROS and TNFα production and increased phagocytic activity. Insulin treatment also significantly reduced iNOS expression, but had no significant effect on any other M1 or M2 macrophage polarization marker examined. These data suggest that insulin has very specific effects to reduce pro-inflammatory or chemoattractant properties of microglia, and this may be one mechanism by which insulin has beneficial effects in CNS injury or neurodegenerative conditions.

## Introduction

Microglia are resident immune cells of the brain responsible for sensing and maintaining homeostasis.[1–3] In their quiescent state, microglia sample the surrounding environment with their long processes.[3] Microglial activation occurs in response to infection, injury, inflammation, neuronal cell death and cytokine release.[4] Microglia respond to a broad spectrum of stimuli including tumor necrosis factor α (TNFα), interleukin 6 (IL-6), and interleukin 1β (1IL-1β) as well as lipopolysaccharide (LPS), a bacterial cell wall product.[5] Regardless of the source of stimulus, microglial activation results in a series of well–documented outcomes, including changes to cellular morphology including shortened processes and enlarged cell body.[6]

Microglial activation occurs along a spectrum that ranges from pro-inflammatory activation to anti-inflammatory activation.[5, 7, 8] Pro-inflammatory microglia typically produce nitric oxide (NO), reactive oxygen species (ROS) and a number of pro-inflammatory cytokines.[9] NO plays an important role as a messenger but its prolonged release from microglia can be detrimental, as NO and its degradation products are highly reactive and cause DNA deamination and neuronal cell death.[10–12] NO release is mediated by the expression of inducible NO synthase (iNOS).[13, 14] Production of ROS promotes inflammation and increased extracellular release over time overwhelms the antioxidant systems of neurons resulting in lipid peroxidation, oxidative protein modifications and cell death.[15, 16] Additionally, microglia release pro-inflammatory cytokines, such as TNFα, that draw more microglia to the site, increasing the inflammatory response. The anti-inflammatory phenotype, on the other hand, produces anti-inflammatory cytokines and can be neuroprotective.[17, 18]

Microglial activation is an important aspect of the pathology of trauma and neurodegenerative diseases, such as traumatic brain injury and Alzheimer’s disease. Additionally, dysfunction of the insulin receptor has been implicated in the pathology of Alzheimer’s disease.[19, 20] Recent studies have shown that insulin administered directly to the brain via intranasal drug delivery can improve cognitive deficits associated with Alzheimer’s disease and aging.[21, 22] A significant reduction in microglia was observed following intranasal insulin treatment in a mouse model of Alzheimer’s disease.[23] Our lab has shown a significant improvement in memory function after moderate brain injury with intranasal insulin treatment.[24] Interestingly, this improvement in memory function was correlated with a significant reduction in microglial staining in the brain after intranasal insulin treatment, similar to that observed by Chen et al.[23]

Only one study to date has directly examined the effect of insulin on microglia, utilizing a human microglia cell line.[25] This work showed that insulin administration significantly reduced the production of monocyte chemoattractant protein-1 (MCP-1) but increased interleukin-8 (IL-8) release in human microglia cultures stimulated with a combination of IL-6, TNF-α and IL-1β. This resulted in a significant reduction in microglial-induced toxicity to neuronal cells. However, the effect of insulin on microglial polarization and oxidative stress has not yet been established. Therefore, the purpose of this study was to examine the effect of insulin on activated microglia *in vitro*. This study verifies that insulin has an anti-inflammatory effect on the BV2 microglial cell line, reducing NO, ROS and cytokine production and increasing phagocytic activity, which provides further support to the use of insulin in the treatment of Alzheimer’s disease and neurotrauma.

## Methods

### Study design

The BV2 microglia cell line (a gift from Carol Colton, Duke University) was cultured in a humidified incubator at 37°C 5% CO_2_/95% oxygen. BV2s are an excellent model of primary microglia inflammatory response following LPS stimulation.[26] The cells were maintained with Dulbecco’s Modified Eagle Media (DMEM, Invitrogen) with 10% fetal bovine serum (FBS, Hyclone, Logan, UT, USA) and 1% penicillin/streptavidin (Fisher, Pittsburgh, PA, USA). BV2s were extracted from flasks with Accutase (Innovative Cell Technologies, San Diego, CA) after reaching confluency and plated at 1X10^5^ density for all experiments. Each outcome measure was repeated in triplicate.

### Treatments

BV2 cells were treated with a 125ng/ml concentration of lipopolysaccharide (LPS) or a 50ng/ml concentration of TNFα (R&D Systems) one hour prior to insulin treatment. Commercially available Humulin R (Eli Lilly Indianapolis, IN) was administered to BV2 cells with a concentration range of 0.09μM, 0.18μM, 0.36μM, 0.72μM, and 1.45μM for 24 hours.

### Nitric oxide measurement

NO was measured in BV2s treated with insulin after LPS activation and a 24 hour incubation period. NO content in the media was quantified using a Griess reagent assay kit (Invitrogen). The assay was performed per manufacturer’s recommendations and as previously described.[27, 28] Colorimetric changes in 96-well plates were quantified with a Chroma Plate Reader (Midwest Scientific, St. Louis, MO, USA) at 545 nm.

### Reactive oxygen species measurement

ROS production following each treatment paradigm was quantified per the manufacturer’s recommendations and as previously described.[27] The assay measures the oxidation of 5 (and 6)-chloromethyl-20,70-dichlorodihydro-fluorescein diacetate-acetyl ester (CM-H2DCFDA; Molecular Probes, Eugene, OR). Briefly, media from microglia plated into 96-well plates was removed and replaced with warmed PBS. CM-H2DCFDA (10 μM) was added to microglia and incubated for 45 minutes. Fluorescence was measured using excitation wavelengths of 492–495 nm and emission wavelength of 517-527nm.

### Immunocytochemistry

Cells were plated on coverslips and fixed with 4% paraformaldehyde at 24 hours for immunocytochemistry analysis. Cells were incubated with blocking agent for 15 minutes then incubated overnight with primary antibody. The primary antibodies for analysis of microglia activation were CD206 (1:500,Abcam) and iNOS (1:500, Cell Signaling,). Slides were incubated for one hour with a secondary fluorescent antibody (Alexa-Fluor Secondaries, Invitrogen, Grand Island, NY) for one hour then coverslipped with hard set DAPI mounting media (Vectashield, Vector Laboratories, Inc., Burlingame, CA) Five images at 20X magnification were collected from each coverslip. Density of immunolabeled protein above a threshold standardized between coverslips was normalized to number of DAPI positive cells, obtained using ImageJ (http://imagej.nih.gov/ij/, NIH, Bethesda, MD).[29]

### Protein quantification

Protein was collected from cells at 2 or 24 hours post treatment. Cells were lysed, treated with protein inhibitor (Halt Protease Inhibitor single use cocktail, Thermo Scientific) then centrifuged. The supernatant was collected and western blot analysis was performed as previously described. [30] Briefly, 25 μg of protein sample was run on a Mini-protean TGX precast gel then transferred to a nitrocellulose sheet (Trans-Blot Turbo Transfer System, Biorad, Hercules, CA, USA). The nitrocellulose sheet was blocked for one hour then incubated overnight with the following antibodies: p-AKT (Thr308, 1:1000, Cell Signaling), AKT (1:1000, Cell Signaling), p-AKT (ser473, 1:1000, Cell Signaling), iNOS (1:500, Abcam), NOX2 (1:500, Abcam), CD86 (1:5000, Abcam), and YM1 (1:1000, Cell Signaling). Resultant bands were quantified with Image J densitometry and normalized to a loading control protein, GAPDH (1:5000, Millipore, Billerica, MA, USA).

### Oxyblot

The OxyBlot Protein Oxidation Detection Kit (Millipore) was conducted per the manufacturer’s instructions. Briefly, protein samples were treated with 1X 2,4-Dinitrophenylhydrazine (DNPH) Solution followed by a neutralization solution for derivatization reaction. A protein sample for each specimen was not subjected to derivatization reaction and thus served as a control. Samples were then run on a mini-protean TGX precast gel and then transferred to a nitrocellulose sheet (Trans-Blot Turbo Transfer System, Biorad, Hercules, CA, USA). After one hour blocking at room temperature, the nitrocellulose sheet was incubated for one hour with the primary antibody, after which it was washed and incubated with secondary antibody. The sheet was developed and the entire row was taken per sample for quantification using Image J densitometry. Samples were normalized to the GAPDH loading control protein (1:5000, Millipore, Billerica, MA, USA.).

### ELISA

ELISAs for TNFα (eBioscience, Waltham, MA) and IL8 (MyBioSource, San Diego, CA) were performed on cell supernatant obtained at 24 hours after LPS and insulin addition. Kits were performed according to the manufacturer’s instructions and quantified with a Chroma Plate Reader (Midwest Scientific, St. Louis, MO, USA), with protein concentration obtained from the provided standard dilution curve.

### Phagocytosis assay

At 24 hours after incubation with insulin and/or LPS, cells were assessed for phagocytic activity using the Phagocytosis Assay Kit (Cayman Chemical, Ann Arbor, MI) or the Vybrant Phagocytosis Assay (Invitrogen). Briefly, for the Cayman Chemical assay, latex beads with rabbit IgG-FITC conjugates (1:100) were incubated with BV2 cells for 4 hours followed by a 1 minute incubation with trypan blue to quench non-phagocytosed bead fluorescence and cell fixation with 4% paraformaldehyde. Cells were counterstained with DAPI and five images at 20X magnification were collected from each coverslip. Pixel density of FITC labeled beads above threshold standardized between coverslips was normalized to number of DAPI positive cells, obtained using ImageJ. This assay was repeated in quintuplicate. The Vybrant assay was performed according to the manufacturer’s instructions at 2 hours after incubation with insulin and/or LPS and TNFα. This assay was repeated in triplicate and assessed using a plate reader for fluorescence.

### Statistical analysis

Statistical analysis was conducted using Graphpad Prism Software version 6.01. (GraphPad Software, San Diego, CA). NO and ROS assays were conducted in triplicate for each trial, and the trials were replicated 3 times to generate a sample size of n = 3 per group. A two way ANOVA with Sidak’s multiple comparisons test was conducted on the averages of each trial for NO, ROS, western blot and immunocytochemistry assay measures. ELISA results were analyzed using a two way ANOVA with Tukey’s post test. For all statistical tests described, a p value < 0.05 was considered statistically significant. Data is presented as mean +/- standard error of the mean (SEM).

## Results

### Insulin administration activates the Akt pathway in BV2 cells

**Fig 1:**
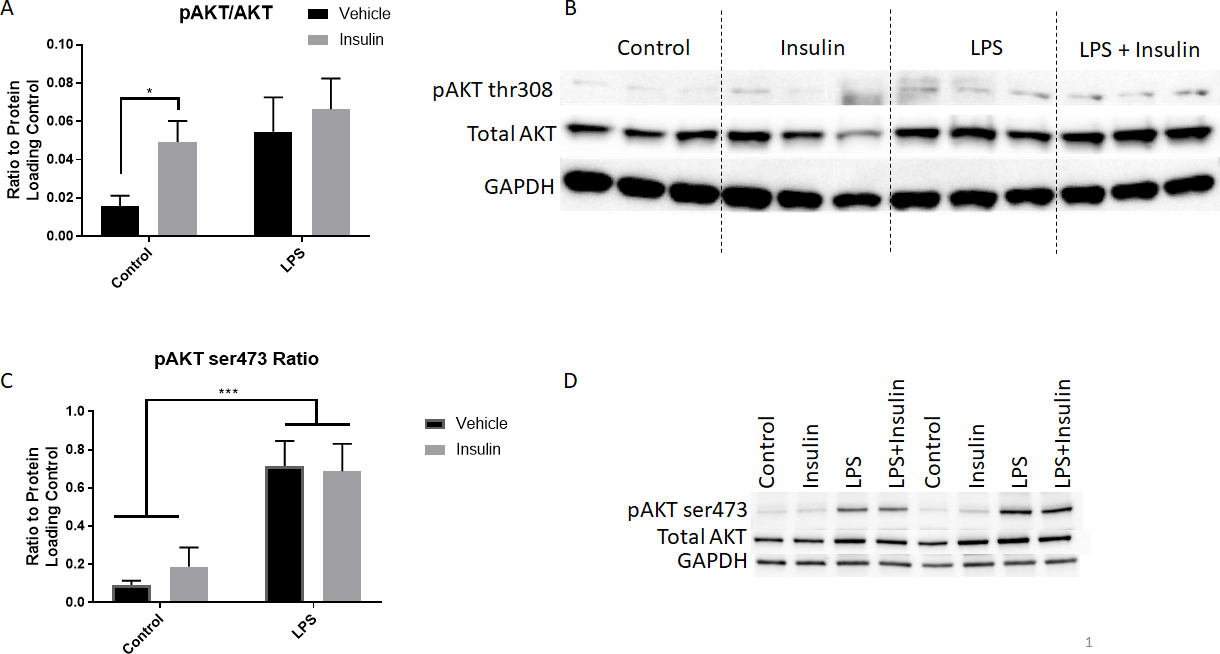
Insulin administration increases AKT thr308 phosphorylation in BV2 cells. Insulin (0.36uM) was administered 1 hour after LPS to BV2 cells and AKT phosphorylation at thr308 or ser473 was assessed 2 hours later. A) Insulin alone significantly increased AKT thr308 phosphorylation. LPS also led to an increase in phosphorylation, but this was not significantly different than the control group. Insulin in addition to LPS led to no significant change in AKT phosphorylation from control or the LPS alone group. *p<0.05, two-way ANOVA. B) Western blot for all samples. C) Phosphorylation of Akt at ser473 was markedly different after insulin and LPS treatment, wherein insulin resulted in a slight but not statistically significant elevation, but LPS significantly elevated phosphorylation. ***p<0.001, two-way ANOVA. N = 3/group. Bars represent mean +/- SEM. D) Representative western blot for 2 of 3 samples for ser473.

To confirm that insulin is acting through the insulin receptor and via the Akt pathway in BV2 cells, we assessed phosphorylation of Akt at ser473 and thr308 2 hours after insulin administration. In BV2 cells that received insulin at 0.36μM, Akt phosphorylation at thr308 was significantly elevated (Fig 1A, two way ANOVA, Sidak’s multiple comparisons post-test). LPS administration also slightly increased Akt thr308 phosphorylation, although this did not reach statistical significance. Insulin in addition to LPS did not significantly alter Akt thr308 phosphorylation in comparison to the LPS or control groups. Phosphorylation of Akt at ser473 was slightly elevated by insulin administration, although this did not reach statistical significance (Fig 1C). LPS, in contrast, significantly elevated ser473 phosphorylation (two-way ANOVA with Sidak’s multiple comparisons post-test). Insulin in combination with LPS led to no further alteration in phosphorylation.

### Insulin treatment significantly reduces NO production

**Fig 2:**
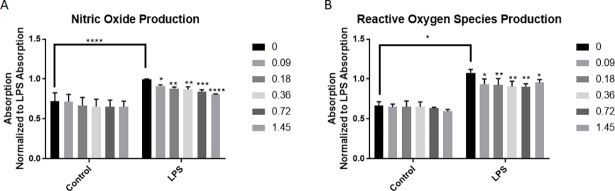
Insulin treatment significantly reduces NO and ROS production. BV2 microglia incubated with LPS showed a significant increase in NO production (A). Insulin treatment after LPS was able to significantly reduce NO production in BV2 microglia. Insulin alone did not significantly alter NO release compared to control cells (0μM insulin) at any concentration. B) BV2 microglia incubated with LPS showed a significant increase in ROS production. Insulin treatment after LPS was able to significantly reduce ROS production in BV2 microglia. Insulin alone did not significantly alter ROS release compared to control cells (0μM insulin) at any concentration. All experimental conditions were repeated in triplicate. *p < 0.05; **p<0.01; ***p<0.001; ****p<0.0001. Bars are mean +/- SEM.

To examine the effects of insulin treatment on activated microglia, BV2 microglia were activated with LPS (125μg) for one hour. Following this incubation, cells were treated with insulin (0.09μM, 0.18μM, 0.36μM, 0.72μM, or 1.45μM). NO and ROS assays were conducted 24 hours after the addition of insulin. Control cells not treated with LPS were treated with insulin at the same time with the same concentrations. Insulin alone did not significantly affect NO release (Fig 2A). LPS treatment significantly increased NO release compared to control (Fig 2A; p = 0.0467, two way ANOVA, Sidak’s multiple comparisons post-test). However, insulin was able to significantly reduce NO production in BV2 microglia following LPS activation in a concentration dependent manner (two way ANOVA, Sidak’s multiple comparisons post-test).

LPS incubation also significantly increased the production of ROS in BV2 microglia compared to control (Fig 2B; p<0.0001, two way ANOVA, Sidak’s multiple comparisons post-test). BV2 microglia treated only with insulin did not show any significant difference in ROS production compared to control. Insulin at all concentrations significantly reduced ROS production in BV2 microglia exposed to LPS (two way ANOVA, Sidak’s multiple comparisons post-test).

### Insulin treatment significantly alters expression iNOS, but not other pro- or anti-inflammatory markers

**Fig 3:**
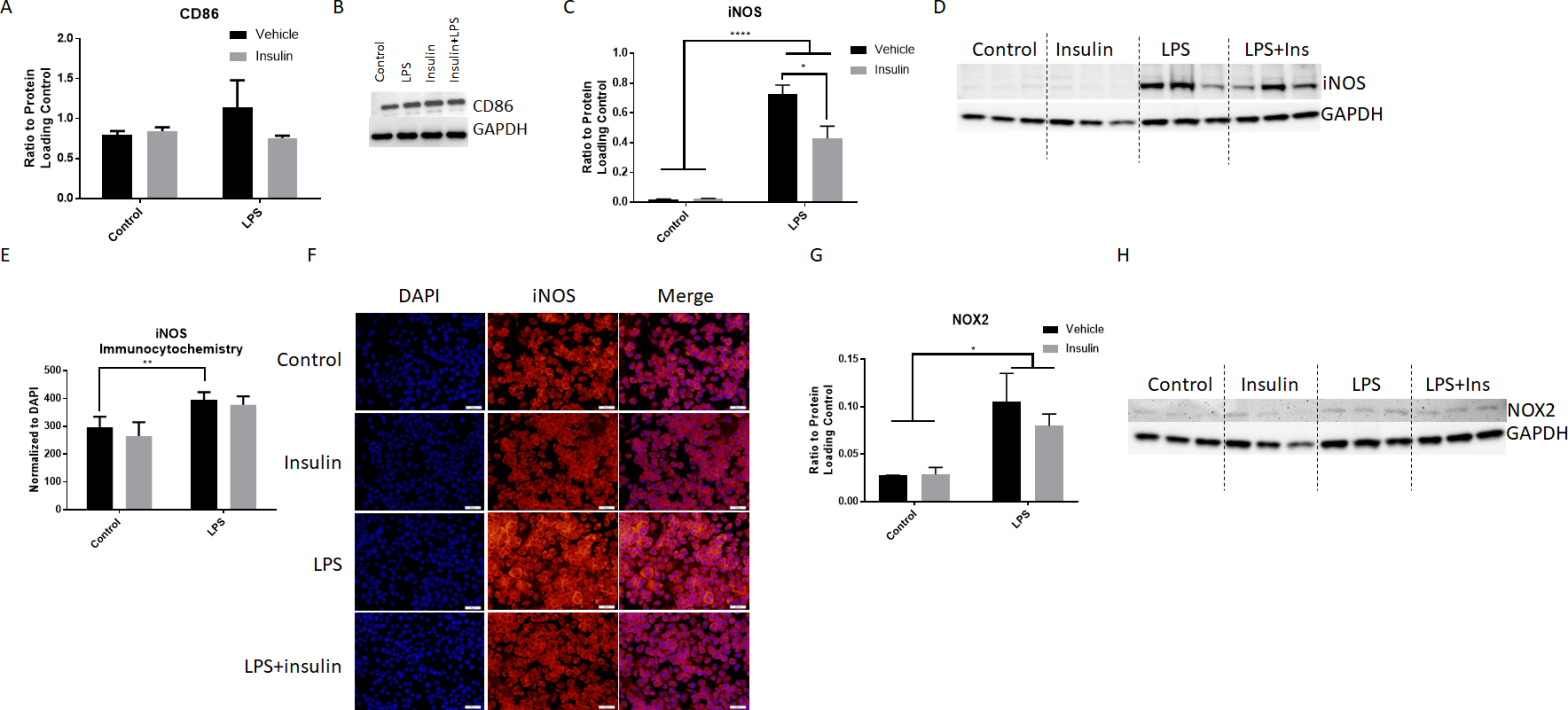
Insulin treatment reduces the expression of iNOS, but not other pro-inflammatory protein markers. Western blot quantitation of CD86 showed no significant difference with LPS or insulin administration (A; n = 3/group). LPS exposure resulted in a non-significant increase in CD86 expression. B) Representative blot of CD86 and GAPDH. Western blot quantitation of iNOS showed a significant elevation in iNOS with LPS administration, and this was significantly blunted by insulin (C, n = 3/group). Blots of the quantified bands are shown in D, with ladder for appropriate band sizing shown at each edge. Quantitation of iNOS immunocytochemistry normalized to DAPI expression showed a significant increase with LPS (E), but, while the image shows a slight reduction iNOS intensity (F), this was not reflected in pixel density quantitation (n = 5-6/group). Western blot quantitation of NOX2 showed a significant elevation with LPS administration, this was not significantly blunted by insulin (G, n = 3/group). Blots of the quantified bands are shown in H. Bars are mean +/- SEM. *p<0.05; **p<0.01; ****p<0.0001. Size bar = 50μm.

Microglial activation was further assessed by the quantification of several well-established markers. iNOS and CD86 are associated with pro-inflammatory activation of microglia. Following one hour LPS activation, BV2 microglia were treated with insulin. The expression of CD86 was quantified using western blot and normalized to GAPDH (Fig 3A and 3B). LPS treatment resulted in a non-significant increase in CD86 expression (Fig 3A). Insulin treatment did not affect CD86 expression in either LPS treated or control cells.

iNOS expression was detected using both western blot and immunocytochemistry. Quantitation of western blot showed that LPS significantly increased iNOS expression at 24 hours after incubation (p<0.0001, two way ANOVA, Sidak’s multiple comparisons post-test; Fig 3C). Addition of insulin 1 hour after LPS administration significantly reduced this iNOS expression (p = 0.012, two way ANOVA, Sidak’s multiple comparisons post-test; Fig 3C). Imaging of iNOS showed a marked increase in immunolabeling following LPS addition (Fig 3F), which was slightly reduced with insulin administration. However, quantitation of pixel intensity normalized to total number of DAPI positive cells showed a significant increase with LPS (p = 0.0099, two way ANOVA, Sidak’s multiple comparisons post-test; Fig 3E), but no change with insulin.

The ROS producing enzyme and pro-inflammatory marker NADPH oxidase 2 (NOX2) was next investigated using western blot. Quantitation of western blot showed that LPS significantly increased NOX2 expression at 24 hours after incubation (p<0.038, two way ANOVA, Sidak’s multiple comparisons post-test; Fig 3G). Addition of insulin 1 hour after LPS administration did not significantly reduce this expression, although a slight reduction was observed (Fig 3H).

**Fig 4:**
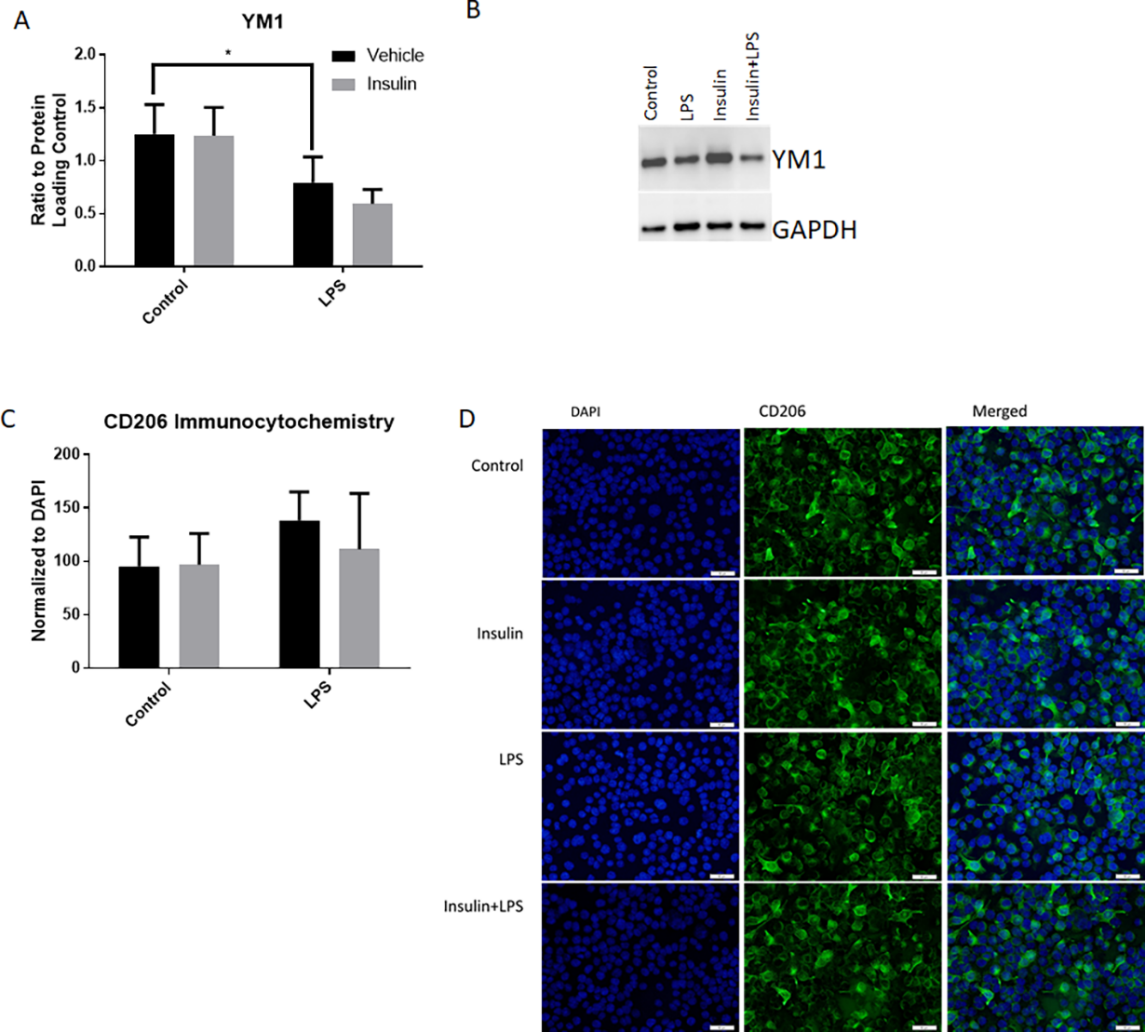
Insulin treatment does not alter expression of anti-inflammatory markers YM1 and CD206. Quantitation of western blots for YM1 showed that LPS exposure significantly decreased expression, but insulin had no effect (n = 3/group, A). Representative bands of YM1 and GAPDH are displayed (B). Quantitation of CD206 immunocytochemistry and normalization to DAPI expression demonstrated no significant effect by any treatment (C). Representative images are shown in D. Bars are mean +/- SEM. *p<0.05. Size bar = 50μm.

The expression of two anti-inflammatory markers, CD206 and YM1, was quantified following insulin and LPS treatment. YM1 expression was quantified using western blot (n = 3 per group). LPS led to a significant increase in YM1 expression (p = 0.0493, two way ANOVA, Sidak’s multiple comparisons post-test between LPS and control; Fig 4A). Insulin led to no significant change in YM1 expression.

Immunocytochemistry for CD206 was quantified following a 24 hour incubation (n = 5 per group for control, insulin alone and LPS, n = 4per group for LPS+insulin, representative images shown in Fig 4D). CD206 expression was not altered in any group in comparison to control (Fig 4C).

### Insulin treatment reduces the release of pro-inflammatory cytokine TNFα

**Fig 5:**
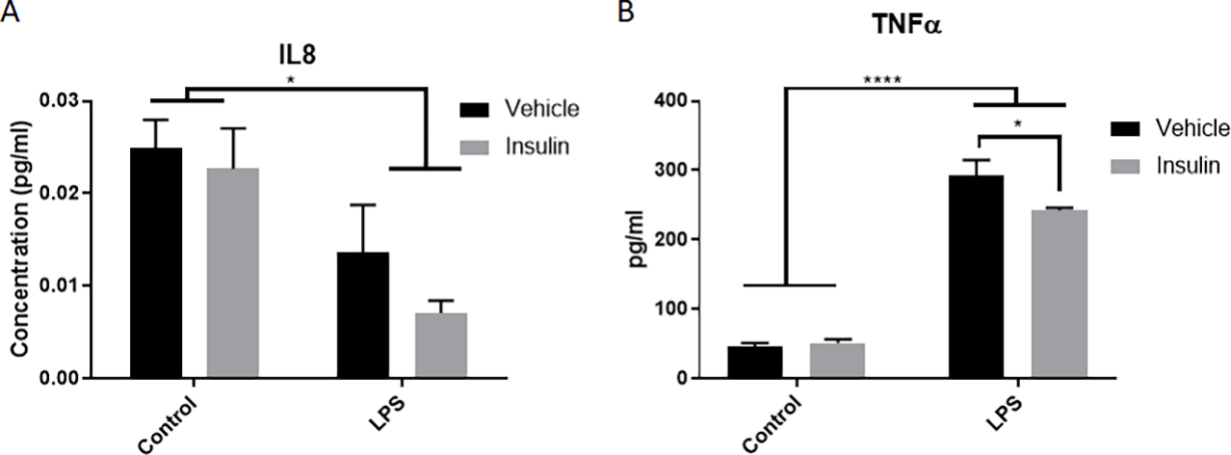
Insulin treatment reduces TNFα. Analysis of chemoattractant and cytokines known to be affected by LPS and insulin showed that, while LPS significantly reduced IL8 production, insulin did not significantly alter this expression. However, insulin did significantly reduce TNFα expression in comparison to the LPS-treated cells. *p<0.05, ****p<0.0001. n = 3/group. Bars represent mean +/- SEM.

A pro-inflammatory cytokine and a chemokine were assessed at 24 hours after insulin and LPS incubation. Samples were collected following a 24 hour incubation (n = 3 per group). LPS significantly reduced release of the IL8 chemoattractant (p = 0.0076, 2 way ANOVA, Sidak’s multiple comparison’s post-test, Fig 5A), but this was not significantly altered by insulin administration. LPS also significantly increased expression of TNFα (p<0.0001, 2 way ANOVA, Sidak’s multiple comparison’s post-test, Fig 5B). This elevation with LPS was significantly reduced by administration of insulin (p = 0.013, 2 way ANOVA, Sidak’s multiple comparison’s post-test, Fig 5A).

### Insulin treatment increases phagocytic activity after LPS stimulation

**Fig 6:**
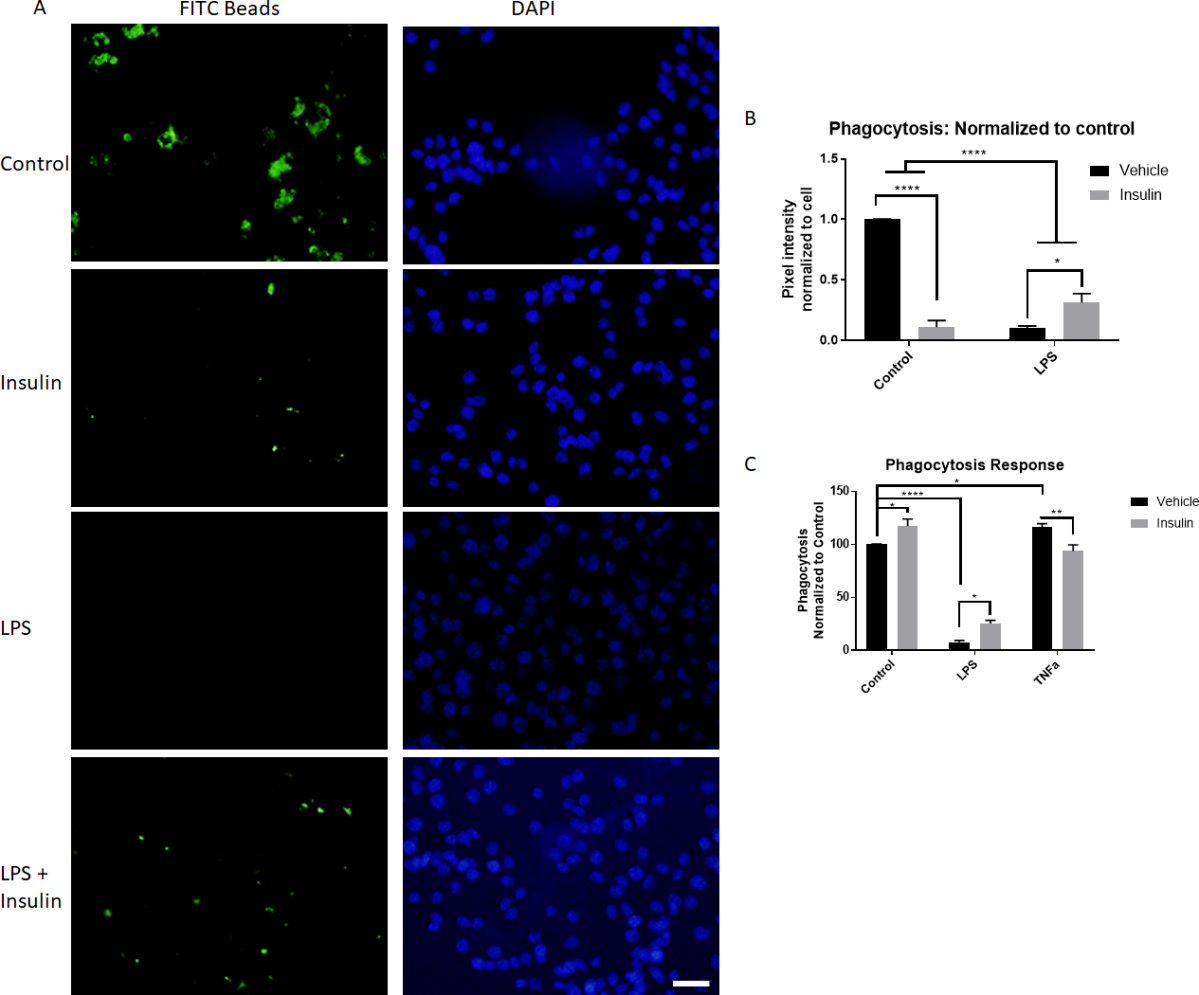
Insulin treatment alters phagocytic activity. A) Latex beads conjugated with FITC-IgG (green) were incubated for 4 hours with BV2 cells (DAPI nuclear stain–blue) exposed to control conditions, insulin, LPS, or LPS + insulin (representative images). B) Quantitation of phagocytosed beads demonstrated that insulin alone led to a significant reduction in phagocytosis activity at 28 hours (24 hour incubation with stimulation, 4 hour incubation with beads), which was further reduced by LPS stimulation. Addition of insulin to the LPS treated group partially alleviated this suppression, significantly increasing phagocytosis in comparison to the LPS group. n = 5/group. C) Vybrant phagocytosis assay performed 3 hours after stimulation (2 hours with phagocytic material), showed that acute insulin administration significantly increased phagocytosis. Similar to the longer condition, LPS reduced phagocytosis, which was reversed by insulin administration. TNFa, on the other hand, significantly increased phagocytosis, which was reversed by insulin. N = 3/group. Bars are mean +/- SEM. *p<0.05, **p<0.01, ****p<0.0001. Size bar = 50μm.

Finally, to assess the phagocytic activity of microglial cells in response to stimulation and insulin administration, BV2 cells were cultured with LPS and/or insulin for 24 hours followed by measurement of phagocytosis of FITC-IgG conjugated latex beads. Surprisingly, insulin administration significantly reduced phagocytosis in comparison to control (p<0.0001, 2 way ANOVA, Sidak’s multiple comparison’s post-test, Fig 6). The addition of LPS similarly reduced phagocytosis in comparison to control (p<0.0001, 2 way ANOVA, Sidak’s multiple comparison’s post-test). Insulin when added to the LPS group at 1 hour after LPS stimulation significantly alleviated this suppression of phagocytosis (p = 0.01, 2 way ANOVA, Sidak’s multiple comparison’s post-test).

To determine if this surprising effect of insulin was due to the prolonged nature of the incubation, we assessed the phagocytic activity of BV2 cells 1 hour after stimulation with insulin and LPS, and added the known phagocytosis inducer TNFα. After the 1 hour incubation, cells were exposed to *E*.*coli* using the Vybrant Phagocytosis assay. With this regimen, insulin administration significantly increased phagocytosis in comparison to control (p = 0.033, 2 way ANOVA, Sidak’s multiple comparison’s post-test, Fig 6C). The addition of LPS continued to reduce phagocytosis in comparison to control (p<0.0001, 2 way ANOVA, Sidak’s multiple comparison’s post-test). Insulin when added to the LPS group at 1 hour after LPS stimulation significantly alleviated this suppression of phagocytosis (p = 0.05, 2 way ANOVA, Sidak’s multiple comparison’s post-test). TNFα, on the other hand, significantly increased phagocytosis (p = 0.01, 2 way ANOVA, Sidak’s multiple comparison’s post-test); this increase was reduced by the addition of insulin (p = 0.007, 2 way ANOVA, Sidak’s multiple comparison’s post-test).

## Discussion

This study provides important information on the inflammatory response of BV2 microglia in response to insulin. This research is relevant to both brain trauma and Alzheimer’s disease research as microglia activation and insulin dysregulation have been implicated in their pathology. Our previous work has demonstrated that insulin can reduce inflammation in the injured hippocampus, however, it was unclear if this action was direct upon microglia. Our data now shows that insulin treatment, acting potentially via the Akt pathway, can reduce NO and ROS production and alter phagocytic activity. In conjunction with these results, we show a significant reduction in iNOS expression, although no significant change in the expression of the ROS producing enzyme NOX2, or other pro- or anti-inflammatory markers was observed, suggesting that insulin’s actions on microglia are not broadly anti-inflammatory.

Activation of the Akt signal transduction pathway involves phosphorylation at threonine 308 and serine 473. Threonine 308 (thr308) lies within the activation loop of Akt and is required for Akt activation; ser473 phosphorylation is required for full activation [31]. We now show that in BV2 microglia, thr308 is phosphorylated at 2 hours after insulin and LPS administration, more so than ser473. LPS led to an increase in ser473 phosphorylation more so than thr308, suggesting different pathways of activation. However, insulin and LPS did not have any effect on each other, in agreement with previous work demonstrating that the Akt pathway is not how insulin modulates LPS induced changes in inflammation [32]. Future work is needed to more fully understand the insulin signaling pathways within microglia.

Previous studies have examined the effect of insulin on the inflammatory response in the periphery. Insulin treatment has been shown to reduce the production of NO and iNOS expression in peripherally circulating macrophages in a rodent model of diabetes [33]. Our study shows that insulin treatment has a similar effect on microglia, the macrophages of the CNS. We show a significant decrease in NO production following LPS stimulation with 0.36μM concentration of insulin (Fig 2A), which correlated with a reduction in iNOS expression.

NO plays an important role in normal function but the excess amount produced as a result of prolonged microglia activation can be detrimental [34]. NO contributes to protein tyrosine nitration, a post translational modification that damages protein function. Excess NO production can result in the nitration of heat shock protein (HSP) 90, which results in cell death [35, 36].

There was also a significant reduction in ROS production in LPS stimulated BV2 microglia treated with insulin (Fig 2B). Evaluation of one enzymatic source of ROS, NOX2, was performed, but while a slight reduction in expression was observed, this did not reach statistical significance. It is important to note, though, that expression of NOX2 is not necessarily correlated with function of the enzyme, and future work will more fully examine the influence of insulin on NOX enzymatic function. Previous work has shown that insulin reduces ROS production in white blood cells [37] although this has never been investigated in microglia. Further, it has been shown that MCP-1 deficiency reduces ROS[38]; as we saw a significant reduction in MCP-1 expression after insulin administration, this may be another means by which insulin affects microglia.

It is worthwhile to note that LPS is a particularly strong activator of microglia that resulted in a significant increase in ROS production compared to controls (Fig 2B). Different activators of microglia like tumor necrosis factor alpha (TNF-α) or IL-6 may produce an activity level that is more amenable to insulin treatment. This was shown in the work of Spielman et al, which showed a significant reduction in IL-8 production as well as MCP-1, but ROS were not measured [25].

Additional insight about inflammatory activity was gained from the ELISA assessments of IL8 and TNFα. Insulin alone did not significantly alter the expression of these cytokines compared to control. However, LPS did significantly reduce IL8 and increase TNFα. While insulin had no effect on LPS-induced changes in IL8, it did significantly reduce TNFα release. IL8 is of interest as it can lead to increased phagocytic activity in peripheral inflammatory cells, such as neutrophils [39]. While no study has evaluated the direct effect of IL8 on microglial cells phagocytic activity, this does provide one explanation for the surprising finding of reduced phagocytosis in our BV2 cells with LPS stimulation (Fig 6). Other studies have shown that pro-inflammatory stimulants such as LPS reduce phagocytosis [40]. Interestingly, phagocytosis was found to be reduced with LPS stimulation at both 1 hour and 24 hours of incubation. However, 1 hour incubation with insulin was found to increase phagocytosis, while these cells showed reduced phagocytosis 24 hours later. Such a difference in response over time has not previously been detailed in microglial or in insulin responses, and additional investigation is warranted. Regardless, the traditional inducer of phagocytosis, TNFα [41], significantly elevated phagocytosis as expected acutely after stimulation. This elevation was reduced by insulin, which is unsurprising considering our findings that insulin blocked TNFα release and other pro-inflammatory effects.

In conclusion, much of our study serves to validate findings observed in other models, showing a correlation between insulin treatment and a reduction in NO, ROS and cytokines in macrophages/microglia. This work demonstrates that insulin has direct effects on inflammatory cells in the brain, and may play an important role in the mechanism of action of insulin-based therapies currently being considered for CNS disorders including Alzheimer’s disease and TBI [42, 43]. Together this data validates the use of BV2 microglia as a model of insulin effects on inflammation as these cells behaved similarly to responses observed in studies of human microglia.
